# Thoracoscopic “cut‐through” segmentectomy for small‐sized lung cancer in a deep central location

**DOI:** 10.1111/1759-7714.14721

**Published:** 2022-11-05

**Authors:** Satoshi Takamori, Hiroyuki Oizumi, Jun Suzuki, Kaito Sato, Satoshi Shiono

**Affiliations:** ^1^ Department of Surgery II, Faculty of Medicine Yamagata University Yamagata Japan; ^2^ Department of General Thoracic Surgery Higashiyamato Hospital Higashiyamato Japan

**Keywords:** central lesion, lung cancer, subsegmentectomy

## Abstract

The use of segmentectomy and subsegmentectomy for the management of lung lesions is well established. However, the use of subsegmentectomy for deep seated lesions in the upper lobe is difficult because of sufficient surgical margins. Here, we present a patient whose lung lesion was in a deep central area and at the borders of three segments in the upper lobe of the right lung. We used combined subsegmentectomy (S1b + S3a) video‐assisted thoracoscopic surgery for this small‐sized lung cancer in a deep central location.

## Graphical abstract

We present a combined subsegmentectomy (S1b+S3a) technique for small‐sized lung cancer of deep central location via thoracoscopic surgery. In this type of segmentectomy, the segment was separated like cut‐through roads.

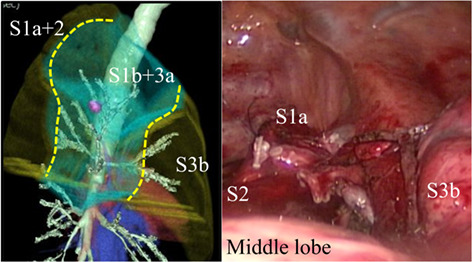

## INTRODUCTION

Favorable results of combined subsegmentectomy and/or segmentectomy for small‐sized lung cancer have been reported to generate favorable outcomes.^1^, ^2^, ^3^ Most clinical studies of segmentectomy focus on its application to peripheral small‐sized lung cancer. Nevertheless, some studies have reported the usefulness of segmentectomy for centrally located lung cancer,^3^, ^4^ and the indication for segmentectomy might expand in the future. However, it is essential to determine whether segmentectomy is difficult for deep lesions in the center of the right upper lobe. Here, we present a case of combined subsegmentectomy (S1b + S3a) for a small‐sized lung cancer of deep central location that was successfully performed via video‐assisted thoracoscopic surgery (VATS).

## CASE REPORT

**FIGURE 1:**
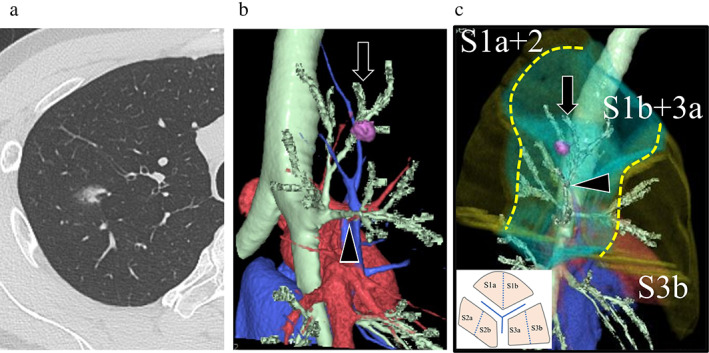
(a) Computed tomography (CT) reveals a lung tumor in the central area of the upper lobe. The tumor distance from the visceral pleura was approximately 3 cm. (b) The tumor is mainly located between B1b and B3a. B1b (arrow) and B3a (arrowhead). (c) We planned combined S1b + 3a segmentectomy using three‐dimensional CT to keep sufficient margins. If sufficient parenchyma is to be preserved, S1bii + S3ai combined segmentectomy should be selected; however, the surgery is very difficult to perform. B1bii (arrow) and B3ai (arrowhead)

A 45‐year‐old man was noted to have a part‐solid ground‐glass nodule (GGN) in the right upper lobe during a physical examination. Preoperative computed tomography (CT) revealed a 1.5 × 1.1 cm tumor (solid component, 0.6 cm) in the S3. Although contrast‐enhanced CT and three‐dimensional CT (3D‐CT) reconstruction with SYNAPSE VINCENT (Fujifilm Medical Co., Ltd.) revealed that the lesion was in a deep central area and at the borders of three segments in the lobe. Considering that the volume of S3 of this patient was larger than usual and S3b preservation would benefit the patient (Figure 1a–c), we planned a right S1b + 3a combined subsegmentectomy. Moreover, the surgical margins were determined to be greater than the tumor diameter using 3D‐CT.

**FIGURE 2:**
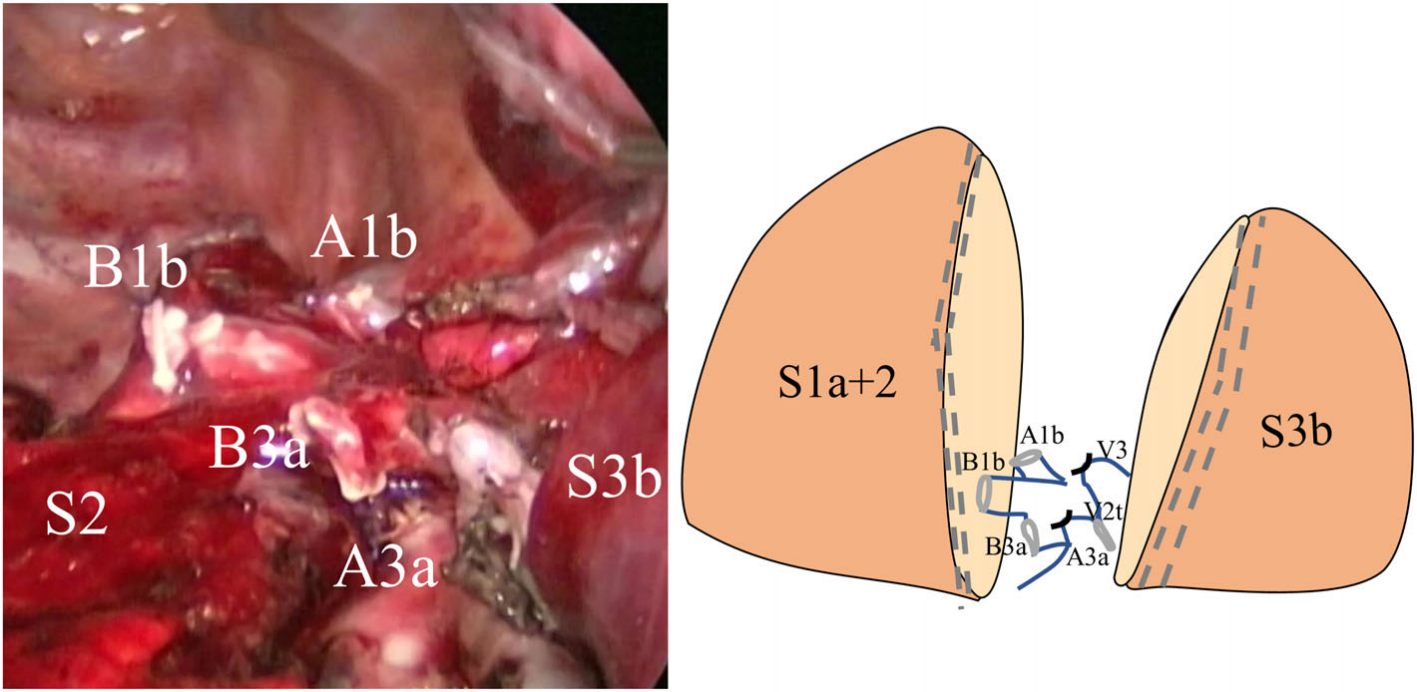
Segmental plane after combined S1b + 3a segmentectomy and schema. The central part of the upper lobe is resected. When the remaining segment was expanded, the remaining segment of the upper lobe became mountainous and looked like a cut‐through road; hence, we named this resection method “cut‐through” segmentectomy

After opening the incomplete fissure between the upper and middle lobe, we identified the A3a, which we divided. Before dividing B3a, we performed the slip‐knot method to create an inflation–deflation line of the segmental plane.^5^ After dividing B3a, we identified A2a, 1a, and 1b by retracting the lung caudally. A1b was clipped and dissected. For B1b, we performed the slip‐knot method again, and then divided it. After releasing the hilar structures, we used staplers to cut the intersegmental planes. The post removal view was similar to the cut‐through road (Figure 2). We sutured the segmental plane to prevent torsion, adhesion, and air leakage. Intraoperative segmental lymph node (no. 13) metastasis was absent; therefore, mediastinal lymph node dissection was omitted. The operation time was 213 min, and the total blood loss was 155 ml. The postoperative course was uneventful, and the patient was discharged 5 days after surgery. Histopathological examination confirmed a papillary adenocarcinoma (tumor diameter: 1.2 cm, solid component: 0.7 cm, papillary component: 50%, lepidic component: 50%, two #13 lymph nodes removed, margin: 1.2 cm, pT1aN0M0‐IA1). We proposed a completion lobectomy; however, the patient refused to undergo additional surgery. The patient has been alive for 5 years postoperatively without recurrence, and the remaining lung in the upper lobe is well inflated.

## DISCUSSION

The indications for anatomical segmentectomy have expanded because it reportedly yields relatively good clinical outcomes.^1^, ^2^, ^3^, ^4^ Our institution has reported comparable surgical and long‐term outcomes for segmentectomy and/or subsegmentectomy.^2^, ^3^ We believe that the approach and resection concept of subsegmentectomy and segmentectomy are basically the same.^2^, ^3^, ^6^ The problem is the higher local recurrence than lobectomy; in particular, segmentectomy reportedly has higher local recurrence for right upper lobe tumors than for those in other lobes.^7^ If the tumor, such as pulmonary metastasis and pure GGN, is located deep in the center of the lobe, lobectomy might be preferred to ensure the adequate margins. We have reported a good outcome of segmentectomy or susegmentectomy even for those in the tricky location.^3^ A segmentectomy for the right upper lobe must be performed cautiously to ensure adequate margins. Even with deep central lesions in the right upper lobe, as in this case, sufficient margins can be planned and performed by combining subsegmentectomy with 3D‐CT.

Essential to the operation is the precise interpretation of the structure of the target lung segment and to review from various angles before the surgery using enhanced CT and 3D‐CT. The key of this procedure is to release the hilar structures and open both intersegmental planes using staplers. The view post‐segmentectomy is similar to a cut‐through road. While there has been a perioperative report of similar method,^8^ this study presented our anatomically combined subsegmentectomy (S1b + 3a) technique for small‐sized lung cancer in the center of right upper lobe. Moreover, the patient had good long‐term results. With this method, both raw cut segment surface fuse together after the operation, and dead space is small because of a large residual lung and this method might prevent air leakage, one of the serious complications of segmentectomy. Further, it might be advantageous in different cases, such as second primary lung cancer, to prevent excess adhesion in the future.

Although subsegmentectomy such as that reported here is rarely indicated, we believe that it has the potential to have a good outcome and we expect that it will be reported in prospective studies.

We demonstrated a combined subsegmentectomy via VATS for a centrally located lung cancer in the right upper lobe. “Cut‐through” segmentectomy that preserves as much parenchyma as possible for deep central lesions in the upper lobe is a feasible technique.

## CONFLICT OF INTEREST

The authors have no conflicts of interest to declare.

## Supporting information


**Video S1.** Combined subsegmentectomy (S1b + S3a) for deeply located small‐sized lung cancer via video‐assisted thoracoscopic surgery.

## References

[1] Saji H , Okada M , Tsuboi M , Nakajima R , Suzuki K , Aokage K , et al. Segmentectomy versus lobectomy in small‐sized peripheral non‐small‐cell lung cancer (JCOG0802/WJOG4607L): a multicentre, open‐label, phase 3, randomised, controlled, non‐inferiority trial. Lancet. 2022;399:1607–17. 35461558 10.1016/S0140-6736(21)02333-3

[2] Kato H , Oizumi H , Suzuki J , Suzuki K , Takamori S . Roles and outcomes of thoracoscopic anatomic lung subsegmentectomy for lung cancer. Interact Cardiovasc Thorac Surg. 2022;34:81–90. 34999803 10.1093/icvts/ivab221 PMC8932510

[3] Takamori S , Oizumi H , Suzuki J , Suzuki K , Kabasawa T . Video‐assisted thoracoscopic segmentectomy for deep and peripheral small lung cancer. Thorac Cardiovasc Surg. 2022;70:233–8. 33540428 10.1055/s-0040-1722172 PMC9192317

[4] Tane S , Kimura K , Shimizu N , et al. Segmentectomy for inner location small‐sized non‐small cell lung cancer: is it feasible? Ann Thorac Surg. 2022;114:1918–24. 10.1016/j.athoracsur.2021.08.035 34563504

[5] Oizumi H , Kato H , Endoh M , Inoue T , Watarai H , Sadahiro M . Slip knot bronchial ligation method for thoracoscopic lung segmentectomy. Ann Thorac Surg. 2014;97:1456–8. 24694436 10.1016/j.athoracsur.2013.07.125

[6] Okada M , Mimura T , Ikegaki J , Katoh H , Itoh H , Tsubota N . A novel video‐assisted anatomic segmentectomy technique: selective segmental inflation via bronchofiberoptic jet followed by cautery cutting. J Thorac Cardiovasc Surg. 2007;133(3):753–8. 17320579 10.1016/j.jtcvs.2006.11.005

[7] Nishio W , Yoshimura M , Maniwa Y , Kitamura Y , Tane K , Takenaka D , et al. Re‐assessment of intentional extended segmentectomy for clinical T1aN0 non‐small cell lung cancer. Ann Thorac Surg. 2016;102:1702–10. 27526651 10.1016/j.athoracsur.2016.05.071

[8] Hong R , Chen C , Zheng W , Zheng B , Xu C , Xu G . ‘Split’ combined subsegmentectomy: a case series. Thorac Cancer. 2022;13:423–9. 34907669 10.1111/1759-7714.14275 PMC8807283

